# Prevalence of Resistance‐Associated Mutations in RSV F Protein Against Monoclonal Antibodies Prior to Widespread Implementation: Findings From a Prospective German Pediatric Cohort

**DOI:** 10.1111/irv.70164

**Published:** 2025-09-18

**Authors:** Martin Wetzke, Duyen Bao Le, Inga Tometten, Simon Ritter, Nadine Lübke, Jörg Timm, Alexander Dilthey, Marcus Panning, Andreas Walker, Christine Happle

**Affiliations:** ^1^ Department of Pediatric Pneumology, Allergy, and Neonatology Hannover Medical School Hannover Germany; ^2^ German Center for Lung Research Biomedical Research in End‐stage and Obstructive Lung Disease Hannover (BREATH) Hannover Germany; ^3^ Institute of Virology, Medical Faculty and University Hospital Düsseldorf Heinrich Heine University Düsseldorf Düsseldorf Germany; ^4^ Center for Digital Medicine Heinrich Heine University Düsseldorf Düsseldorf Germany; ^5^ Institute of Virology, Medical Center, Faculty of Medicine University of Freiburg Freiburg Germany; ^6^ Excellence Cluster on Infection Research “RESolving Infection Susceptibility” RESIST Hannover Germany

**Keywords:** clesrovimab, evasion, mutation, nirsevimab, palivizumab, resistance, RSV

## Abstract

**Background:**

The emergence of resistance‐associated substitutions in RSV against novel monoclonal antibodies is a concern given widespread prophylactic use.

**Aim:**

To assess the prevalence of resistance‐associated substitutions in the RSV F protein against nirsevimab, clesrovimab, and palivizumab in German infants before widespread implementation of nirsevimab.

**Materials & Methods:**

We sequenced the F protein of *n* = 1042 RSV samples from German infants from seasons 2021/2022 and 2022/2023 and screened for variants in binding sites for nirsevimab (Site Ø), clesrovimab (Site IV), and palivizumab (Site II).

**Results:**

Prevalence of resistance‐associated substitutions was low (< 1%) for all three monoclonal antibodies.

**Conclusion:**

Although the current risk of infections with escape‐mutants appears to be low, our results underline the need for continued surveillance, as resistance‐conferring mutations to new mAbs circulated and may be selected under selection pressure.

## 1 Current Situation and Aim

Respiratory syncytial virus (RSV) is a leading cause of infant morbidity and mortality globally [1]. Recently approved monoclonal antibodies, including nirsevimab [2] and clesrovimab [3], represent significant advances in RSV prevention. Nirsevimab received European approval in 2022 and has been widely implemented, with broad coverage, for example, in France and Spain [4], and clesrovimab has recently been approved in the United States [5]. In Germany, the Standing Committee on Vaccination at the Robert Koch Institute (STIKO) recommended the use of nirsevimab for primary prevention of RSV disease in all infants in the first year of life, regardless of the presence of a risk constellation as of June 2024 [6]. As with other antimicrobials and in particular observed for SARS‐CoV‐2, concerns about potential resistance development have been raised for RSV, especially considering the viruses' genetic variability and selective pressure from widespread prophylactic monoclonal antibody (mAb) use [7].

This study aimed to assess the prevalence of known and potential resistance‐associated substitutions (RAS) in the RSV fusion (F) protein against nirsevimab, clesrovimab, and palivizumab in a large, multicentric, prospective cohort of German infants sampled in the last seasons before the widespread introduction of nirsevimab in Germany.

## 2 Methods and Cohort

**FIGURE 1:**
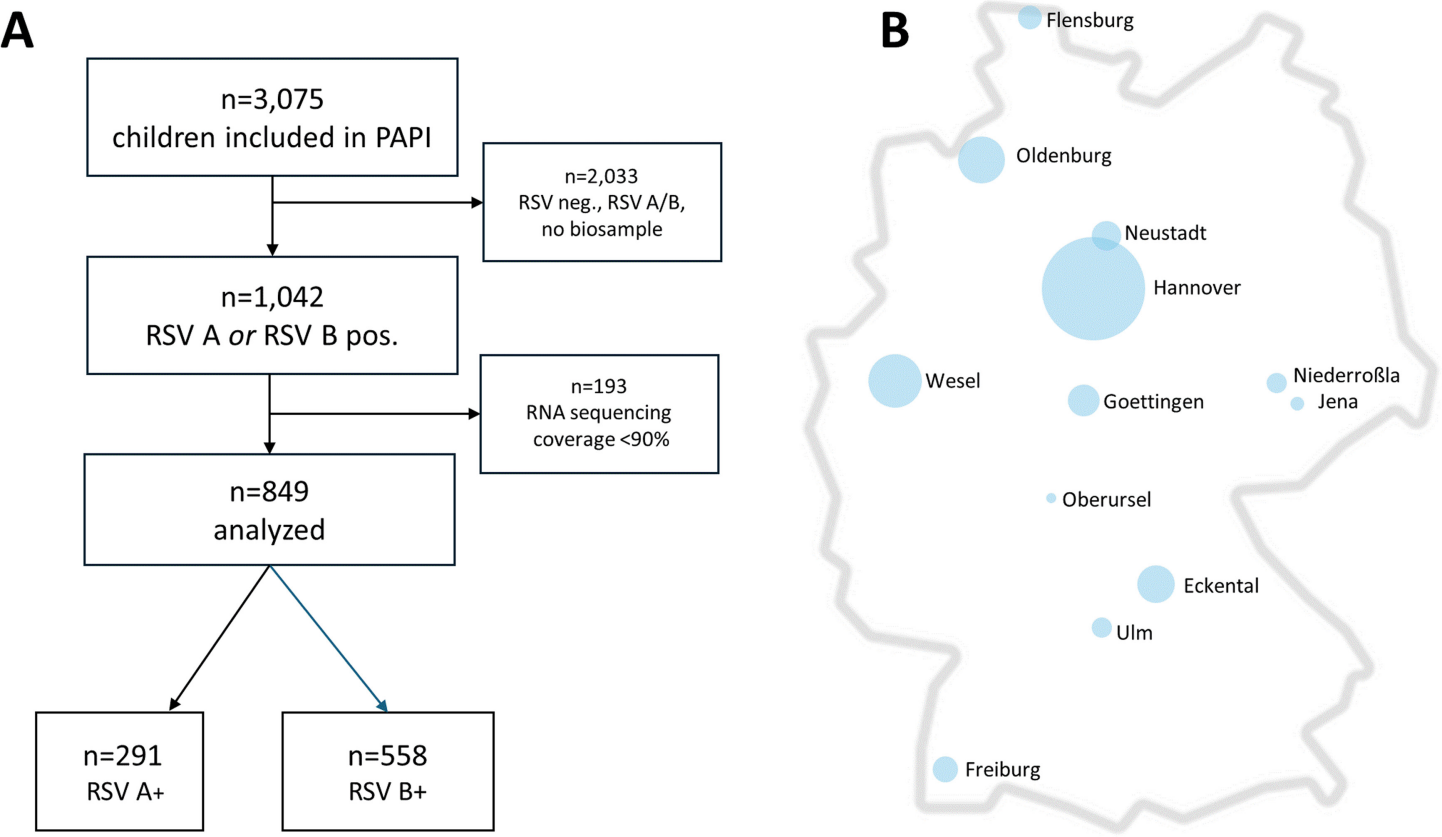
Flowchart of patient inclusion and origin of sequenced virus strains. (A) Inclusion of samples from the PAPI cohort and (B) origin of samples with > 90% of sequencing coverage within Germany.

We analyzed samples from the multicentric, prospective Pediatric Airway Pathogen Incident (PAPI) study, which recruits infants ≤ 24 months of age with lower respiratory tract infections across Germany [8]. The study was approved by the ethics committees of participating centers (Ethical Vote 9442_BO_K_2020 MHH). Legal guardians of each participating child gave their consent. RSV‐A or RSV‐B infections were assessed by PCR (GenMark Diagnostics ePlex Respiratory Pathogen Panel 2, Roche Diagnostics, Rotkreuz, Switzerland, *n* = 1042 children, Figure 1A). Patients were recruited between calendar Week 40 in 2020 and calendar Week 11 in 2023 in hospitals or pediatric practices across Germany (Figure 1B). Less than 0.6% (*n* = 5) of children within this large multicentric cohort had received palivizumab, and in no child, nirsevimab, clesrovimab, or maternal RSV vaccinations had been administered. Virus amplification and sequencing were performed as described previously [9] using the reagents from the NEBNext ARTIC SARS‐CoV‐2 Companion Kit (New England BioLabs), except that SARS‐CoV2 primers were exchanged for pan‐subtype specific RSV primers. Primer sequences and BED files can be found on GitHub (https://github.com/DiltheyLab/RSVTyper/tree/main/rsv_typer). Sequencing data were analyzed employing RSVTyper (https://anaconda.org/bioconda/rsv‐typer) and Nextclade. Sequences with coverage > 90% of the whole genome (*n* = 849) were included in the analysis of mutational patterns (Figure 1A). Of these, *n* = 291 (34.2%) were positive for RSV‐A, and *n* = 558 (65.7%) were positive for RSV‐B.

## 3 Results

From *n* = 1042 RSV samples of the PAPI‐cohort, we excluded *n* = 193 samples due to low sequencing coverage (< 90%, Figure 1A). In the remaining samples, RSV‐B was the most prevalent RSV subtype throughout the overall study period, accounting for approximately two‐thirds of all isolates (Figure 1A). However, predominance differed between seasons (2021/2022: RSV‐A dominance with 71.7%; 2022/2023: RSV‐B dominance with 86.6%). Overall, 74.8% of children were recruited in the outpatient setting (2021/2022: 61.3%; 2022/2023: 82.3%). In around one third of cases (31.3%), viral multiplex analyses showed coinfections, predominantly with rhinovirus. The median age of all study participants was 4 months (IQR 10), and 52.1% of them were male.

First, we analyzed the genetic variation of the RSV F protein RSV‐A and RSV‐B within our cohort. In both strains, RSV F genetic diversity remained low in our samples. The predominant RSV‐B strain was B.D.E.1 (84%), followed by B.D.4.1.1 (14%). All remaining RSV‐B samples belonged to B.D.E.3, B.D.4.1, and B.D.E.4. For RSV‐A samples, a more diverse strain distribution occurred, with a.d.5.2 in 42% of RSV‐A isolates, a.d.1 in 13% of cases and a.d.3 in 13%. All remaining isolates belonged to A. D, a.d.1.4, a.d.1.7, a.d.1.8, a.d.2.1, a.d.3.1, a.d.3.4, a.d.3.5, a.d.5, and a.d.5.3.

Next, we screened for variants in Sites Ø, II, and IV, which confer binding to the mAbs nirsevimab, palivizumab, and clesrovimab (Tables S1–S3).

Analysis of nirsevimab binding Site Ø revealed only sporadic occurrence of amino acid variants' previously described to convey resistance to this preventive mAb. In RSV‐A, one isolate with the variant F:Q202K and another one carrying F:I206T were detected—both with unknown impact on mAb effectiveness. No strain carried previously described resistance mutations.

In RSV‐B, F:S211N was the most common variant, present in approximately 86% of sequences, and 96% of isolates carried the Q209R substitution co‐occurring with I206M (Table 1), which has been lineage defining since B.D.4.1 [10]. These variants have no negative effect on prophylaxis of RSV nirsevimab. In fact, I206M:Q209R increases nirsevimab susceptibility [11]. The F:K68N substitution, known to confer reduced susceptibility to nirsevimab [12], was detected in only one sample (Table 1). The substitutions Q209K/L known to confer partial resistance to nirsevimab [12] were *not* detected. Also, other substitutions previously associated with reduced susceptibility to nirsevimab were absent.

**TABLE 1:** Genetic RSV‐B variants observed in the nirsevimab binding Site Ø (aa62‐69&192‐212).

Isolates (*n* = 558)	S211	R209	K68	M206
64	—	—	—	—
472	N	—	—	—
11	N	Q	—	—
6	—	Q	—	I
4	—	Q	—	—
1	N	—	N	—

For Site II, the palivizumab binding site, in RSV‐A, 26 (9%) of isolates carried a polymorphism F:S276N without impact on palivizumab effectivity [13]. Among RSV‐B isolates, this variant was even less prevalent, and four further substitutions in the palivizumab epitope were observed in low frequencies (Table 2), all of which had unknown impact on antibody neutralization.

**TABLE 2:** Genetic RSV‐B variants observed in the palivizumab binding site.

Isolates (*n* = 558)	F:S276N	F:D263N	F:K271R	F:S275T	F:S276G
552	—	—	—	—	—
1	—	N	—	—	—
1	—	—	R	—	—
2	—	—	—	T	—
1	—	—	—	—	G
1	N	—	—	—	—

The well‐characterized resistance mutations K272E/M/N/Q [14] and N262D [14] were absent in virus isolates of both subtypes across the entire cohort. Of note, none of the isolates from the five RSV‐positive children that had received palivizumab prophylaxis carried any mutations linked to resistance of this mAb.

Regarding clesrovimab, we examined binding Site IV and found only minimal variation. In RSV‐A, we found one isolate (0.3%) G446E, a known resistance variant against this mAb [15]. Another isolate (0.3%) carried K433M, a variant with thus far unknown impact on clesrovimab neutralization. In RSV‐B, three further variants with unclear impact on clesrovimab neutralization effectiveness were identified (F:I432T; F:K445Q; F:V450IF).

When we analyzed chronological effects of time on variation frequency throughout the study period between 2020 and 2023, we observed no significant temporal trends in the frequency of substitutions, and there was no evidence of clustering or spreading of resistant variants.

## 4 Discussion and Conclusion

Our analysis of RSV sequences from a large German cohort revealed a very low prevalence (< 1%) of amino acid variants potentially conferring virus resistance to the mAbs nirsevimab, clesrovimab, and palivizumab.

These results are reassuring, given the recent widespread implementation of nirsevimab prophylaxis in many European countries. In our analysis of RSV‐A, we found no substitutions in Site Ø known to be associated with resistance to nirsevimab, which is in line with a recent paper from France that analyzed virus samples from children with or without nirsevimab prophylaxis during the 2023/2024 season [7]. For RSV‐B, we did not detect substitutions known to confer complete resistance to nirsevimab. However, the population shift at compensatory positions 209 and 206 (Q→R and I→M) suggests that also the F protein is under constant selection pressure. Reversion to previous circulating, potentially resistant variants such as Q209L/K is possible and warrants continued surveillance. Analyzing RSV‐B breakthrough infections, the French team found resistance‐associated substitutions: F:N208D and a newly described F:I64M/F:K65R combination in 8% of cases [2]. Such variants were not observed in our cohort. Of note, however, our cohort was sampled prior to broad introduction of nirsevimab in Germany, and less than 0.6% of children in our cohort had received palivizumab; thus, no selection pressure from broad mAb application was present.

Our study has several limitations. First, we focused on known resistance‐associated substitutions; hence, novel mutations with potential impact on antibody binding may have been missed. Also, we did not perform phenotypic assays to confirm the functional impact of newly observed variants. The selection of infants below the age of two represents only a fraction of children affected by RSV, which infects patients across all ages. However, given the fact that we included patients from the outpatient and hospitalized setting and across all of Germany, we believe our study data do represent the genetic diversity of RSV in the community before the introduction of nirsevimab in this country accurately.

In conclusion, our findings indicate a very low prevalence of mutations conferring resistance to RSV prophylactic mAbs in circulating strains in Germany directly prior to broad implementation of nirsevimab. Our study highlights the importance of ongoing molecular surveillance to monitor the emergence and spread of resistant variants.

## Author Contributions

**Martin Wetzke:** conceptualization, methodology, project administration, writing – original draft, funding acquisition. **Duyen Bao Le:** formal analysis, data curation, writing – original draft, validation. **Inga Tometten:** formal analysis, writing – review and editing, investigation. **Simon Ritter:** investigation, project administration, writing – review and editing. **Nadine Lübke:** formal analysis, investigation, writing – review and editing. **Jörg Timm:** conceptualization, writing – review and editing. **Alexander Dilthey:** writing – review and editing, investigation. **Marcus Panning:** investigation, writing – review and editing, methodology. **Andreas Walker:** conceptualization, writing – original draft, formal analysis. **Christine Happle:** conceptualization, writing – original draft, formal analysis, supervision, visualization.

## Ethics Statement

This study was approved by the ethics committees of the participating centers (vote Medizinische Hochschule 9442_BO_K_2020 MHH). The legal guardians of each participating child were comprehensively informed about the study and gave their written consent. A strobe checklist was added to the manuscript.

## Consent

The manuscript does not contain any identifiable information about individual patients.

## Conflicts of Interest

The PAPI study is supported by funding from Sanofi and AstraZeneca. M.W. has received honoraria for lectures and consultancy work from AstraZeneca, Novartis, Sanofi, Pfizer, and GSK. CH has received research funding from Novartis and Pari, in each case unrelated to the current project.

## Peer Review

The peer review history for this article is available at https://www.webofscience.com/api/gateway/wos/peer‐review/10.1111/irv.70164.

## Supporting information


**Table S1:** Screened variants for clesrovimab/MK‐1654/RB1.
**Table S2:** Screened variants for nirsevimab.
**Table S3:** Screened variants for palivizumab.

## Data Availability

Study data may be made available to third parties with the consent of the PAPI Study Board. RSV sequences used for this study, including associated metadata, are available through GISAID Accession Numbers EPI_ISL_20135429 and EPI_ISL_2013629.
