# Supplementary material for: Prevalence of Resistance‐Associated Mutations in RSV F Protein Against Monoclonal Antibodies Prior to Widespread Implementation: Findings From a Prospective German Pediatric Cohort
Source: Influenza Other Respir Viruses. 2025 Sep 18;19(9):e70164. doi: 10.1111/irv.70164 (PMC12445921; doi:10.1111/irv.70164)
Supplement: Supplementary file 1 — Table S1: Screened variants for clesrovimab/MK‐1654/RB1. Table S2: Screened variants for nirsevimab. Table S3: Screened variants for palivizumab. [file IRV-19-e70164-s001.docx]

**Supplemental Material**

**Suppl. Table 1: Screened variants for clesrovimab/MK-1654/RB1**

| **RSV Subtype** | **Mutation** | **Effect on Susceptibility** | **Source** |
| --- | --- | --- | --- |
| **RSV-A,**  **RSV-B** | G446E | Resistance to neutralization (IC50 > 1000 ng/mL) | Tang et al., 2019 |
| **RSV-A,**  **RSV-B** | S443P | Resistance to neutralization (IC50 > 1000 ng/mL) | Tang et al., 2019 |
| **RSV-A,**  **RSV-B** | G446E/S443P | Resistance to neutralization (IC50 > 1000 ng/mL) | Tang et al., 2019 |
| **RSV-A,**  **RSV-B** | S443P/K445N | Resistance to neutralization (IC50 > 1000 ng/mL) | Tang et al., 2019 |

*Note: MK-1654 differs from RB1 only by the introduction of YTE mutations, which extend serum half-life. MK-1654 targets antigenic site IV, which is highly conserved across both RSV-A and RSV-B strains.*

**Suppl. Table 2: Screened variants for nirsevimab**

| **RSV Subtype** | **Mutation** | **Effect on Susceptibility** | **Source** |
| --- | --- | --- | --- |
| **RSV-B** | N208D | Resistance (>500-fold of IC50 mutant F vs. WT) | Fourati et al., 2025 |
| **RSV-B** | I64M+K65E | Resistance (>500-fold of IC50 mutant F vs. WT) | Fourati et al., 2025 |
| **RSV-B** | K65Q/T | Resistance (25-30-fold increase of IC50) | Zhu et al., 2018 |
| **RSV-B** | Q209R co-occurring with I206M | 0.5-fold resistance (increased effectiveness) | Ahani et al., 2023 |
| **RSV-B** | K68N+N201S | Resistance (>10,000-fold increase) | Zhu et al., 2018 |
| **RSV-A** | E66K | Partial resistance (reported in Iran 2015-2016) | Langedijk et al., 2022 |
| **RSV-B** | K68N | Partial resistance (reported in Kenya, USA 2016-2017) | Langedijk et al., 2022 |
| **RSV-A,**  **RSV-B** | N201S | Partial resistance (reported in South Africa 2015/2017, Netherlands 2017-2018, Korea 2009-2015) | Langedijk et al., 2022 |
| **RSV-A,**  **RSV-B** | Q209K | Partial resistance (reported in China 2014-2016, South Africa 2015, Netherlands 2017-2018, Korea 2009-2015, Philippines 2014-2016, USA 2015-2017) | Langedijk et al., 2022 |
| **RSV-B** | Q209L | Partial resistance (reported in Brazil 2017-2018, USA 2015-2017) | Langedijk et al., 2022 |

**Suppl. Table 3: Screened variants for palivizumab**

| **RSV Subtype** | **Mutation (FASTA)** | **Effect on Susceptibility** | **Source** |
| --- | --- | --- | --- |
| **RSV-A** | S276N | Previously described to confer resistance,  later reported not to impact resistance | Langedijk et al., 2022  Adhikari et al., 2022 |
| **RSV-A** | K272E | Resistance to neutralization (IC50>25000 vs. WT) | Zhu et al., 2011 |
| **RSV-A** | K272N/T/M/Q | Resistance to neutralization (IC50 5000 - >25000 vs. WT) | Zhu et al., 2011 |
| **RSV-A** | S275F/L | Resistance to neutralization (IC50 >25000 vs. WT) | Zhu et al., 2011 |
| **RSV-A** | N262D | Resistance to neutralization (IC50 >100 fold vs. WT) | Zhu et al., 2011 |
| **RSV-A** | N268I | Resistance (1.8 fold reduced vs. WT) | Bates et al., 2014 |
| **RSV-A** | N276S | Resistance (IC50 >100 fold vs. WT) | Adams et al., 2010 |

*Note: S276N in RSV-B samples at the Palivizumab binding site is not associated with resistance. (Langedijk et al., 2022)*

**Supplement references**

*Adams O, Bonzel L, Kovacevic A et al. Palivizumab-resistant human respiratory syncytial virus infection in infancy. Clin Infect Dis. 2010 Jul 15;51(2):185-8. doi: 10.1086/653534.*

*Ahani B, Tuffy KM, Aksyuk AA et al. : Molecular and phenotypic characteristics of RSV infections in infants during two nirsevimab randomized clinical trials. Nat Commun 2023, 14(1):4347.*

*Adhikari B, Hassan F, Harrison CJ et al. A multi-center study to determine genetic variations in the fusion gene of respiratory syncytial virus (RSV) from children <2 years of age in the U.S. J Clin Virol 2022, 154:105223.*

*Bates JT, Keefer CJ, Slaughter JC et al. Escape from neutralization by the respiratory syncytial virus specific neutralizing monoclonal antibody palivizumab is driven by changes in on-rate of binding to the fusion protein.* *Virology. 2014 Apr:454-455:139-44.*

*Fourati S, Reslan A, Bourret J, et al. Genotypic and phenotypic characterisation of respiratory syncytial virus after nirsevimab breakthrough infections: a large, multicentre, observational, real-world study. Lancet Infect Dis 2025; 25: 301–11.*

*Langedijk AC, Harding ER, Konya B, et al. A systematic review on global RSV genetic data: Identification of knowledge gaps. Rev Med Virol. 2022;32(3):e2284.* [*https://doi.org/10.1002/rmv.2284*](https://doi.org/10.1002/rmv.2284)

*Tang A, Chen Z, Cox KS, et al. A potent broadly neutralizing human RSV antibody targets conserved site IV of the fusion glycoprotein. Nat Commun. 2019 Sep 12;10(1):4153. doi:10.1038/s41467-019-12137-1. PMID: 31515478; PMCID: PMC6742648.*

*Zhu Q, McAuliffe JM, Patel NK, et al. Analysis of respiratory syncytial virus preclinical and clinical variants resistant to neutralization by monoclonal antibodies palivizumab and/or motavizumab. J Infect Dis. 2011;203(5):674-682.*

*Zhu Q, Lu B, McTamney P, Palaszynski S et al. Prevalence and Significance of Substitutions in the Fusion Protein of Respiratory Syncytial Virus Resulting in Neutralization Escape From Antibody* *J Infect Dis.. 2018 Jul 13;218(4):572-580. doi: 10.1093/infdis/jiy189.*

*Zhu Q, Patel NK, McAuliffe JM et al. Natural polymorphisms and resistance-associated mutations in the fusion protein of respiratory syncytial virus (RSV): effects on RSV susceptibility to palivizumab. J Infect Dis.. 2012 Feb 15;205(4):635-8. doi: 10.1093/infdis/jir790. Epub 2011 Dec 19*
